# FLCCR is a fluorescent reporter system that quantifies the duration of different cell cycle phases at the single-cell level in fission yeast

**DOI:** 10.1371/journal.pbio.3002969

**Published:** 2025-01-07

**Authors:** Guillem Murciano-Julià, Marina Francos-Cárdenas, Clàudia Salat-Canela, Elena Hidalgo, José Ayté

**Affiliations:** Oxidative Stress and Cell Cycle Group, Universitat Pompeu Fabra, Barcelona, Spain; The Institute of Cancer Research, UNITED KINGDOM OF GREAT BRITAIN AND NORTHERN IRELAND

## Abstract

Fission yeast is an excellent model system that has been widely used to study the mechanism that control cell cycle progression. However, there is a lack of tools that allow to measure with high precision the duration of the different phases of the cell cycle in individual cells. To circumvent this problem, we have developed a fluorescent reporter that allows the quantification of the different phases of the cell cycle at the single-cell level in most genetic backgrounds. To prove the accuracy of this fluorescent reporter, we have tested the reporter in strains known to have a delay in the G1/S or G2/M transitions, confirming the strength and versatility of the system. An advantage of this reporter is that it eliminates the need for culture synchronization, avoiding stressing the cells. Using this reporter, we show that unperturbed cells lacking Sty1 have a standard cell cycle length and distribution and that the extended length of these cells is due to their increased cell growth rate but not to alterations in their cell cycle progression.

## Abstract 2

Current methods to study cell cycle progression in yeast overlook variations in cell size across different strains. This study develops an imaging reporter system called FLCCR that can quantify the duration of each cell cycle phase in single cells and allows the separation of cell size as an independent variable from cell cycle progression.

## Introduction

The cell cycle is a meticulously regulated process governing cellular duplication. At the end of the G1 phase, cells decide whether to commit to a new cell cycle in a pivotal event known as START in yeast or the Restriction Point in higher eukaryotes. Once cells pass this point, they are committed to complete the next cell cycle. The primary regulators of cell cycle progression are cyclin-dependent kinases (CDKs) and their associated cyclins. CDK activity is minimal during the G1 phase but increases in a stepwise manner as the cell progresses through the cycle, with distinct thresholds at the G1/S and G2/M transitions, reaching peak activity at metaphase [1,2].

In fission yeast, early studies of cell cycle progression relied on observations of cell size. Changes in the length of the cell cycle, due to faster or slower progression, result in corresponding decreases or increases in cell size. This relationship led to the identification of classic *cdc* (cell division cycle) and *wee* (small-sized) mutants [3]. These studies also benefited from temperature-sensitive mutants, which allowed synchronization of cells at specific cell cycle phases, enabling synchronous release into subsequent cell cycle stages. Commonly used mutants include *cdc25-22* (a thermosensitive mutant of the CDK-activating phosphatase, which arrests cells at the G2/M transition [4]), *cdc10-129* (a thermosensitive mutant that disrupts MBF-dependent transcription, arresting cells at the G1/S transition [5]), and *nda3-KM311* (a cold-sensitive mutant of the β-tubulin subunit, which prevents microtubule polymerization and arrests cells at metaphase [6]).

While these mutants have been powerful tools, temperature-sensitive mutations introduce limitations. Temperature shifts create stress, impacting cell fitness and potentially introducing artifacts due to non-optimal growth conditions [7]. To address these challenges, ATP analog-sensitive kinase mutants were developed. For example, the *cdc2-M17* allele allows precise inactivation of CDK at the G1/S or G2/M transitions by adding bulky ATP analogs that inhibit kinase activity. These analogs, which compete with ATP for the kinase ATP-binding pocket but cannot be processed, enable fine-tuned control of CDK activity [8]. This approach has facilitated detailed studies of CDK activity thresholds, demonstrating that a single B-type cyclin-CDK chimera can drive the cell cycle [1]. Additionally, it was shown that even an S-phase CDK chimera can fulfill the same role if expressed at high levels or when phosphatase activity is reduced, lowering CDK thresholds [9].

Despite its advantages, the use of bulky ATP analogs is not without drawbacks. Off-target effects, such as the unintended inactivation of the MAP kinase Sty1 by 1NM-PP1 in its wild-type form, can confound results [10]. To overcome such issues, alternative strategies for studying the cell cycle have been developed, including the fluorescence ubiquitin cell cycle indicator (FUCCI) in mammalian cells. FUCCI uses 2 tagged proteins: a red-tagged Cdt1 and a green-tagged geminin. During G1, Cdt1 accumulates, staining cells red. At S phase, Cdt1 is degraded by the SCF complex, and geminin accumulation gives cells a green fluorescence, which persists until metaphase when geminin is degraded via APC/C [11].

However, FUCCI is not applicable to fission yeast, where the G1 phase is extremely short relative to the other cell cycle phases [12]. Furthermore, the degradation of Cdt1 and geminin is influenced by factors beyond cell cycle progression, including DNA damage, stress responses, and protein stability changes, which can obscure the identification of specific cell cycle phases. These limitations highlight the need for innovative tools tailored to the unique characteristics of fission yeast to study cell cycle regulation more precisely.

Here, we have developed a fluorescent-localized cell cycle reporter (FLCCR) for fission yeast that does not rely on new protein synthesis and degradation and that is designed to measure the duration of each cell cycle phase in unperturbed, asynchronous cells, regardless of their genetic background. We have verified its functionality with different known cell cycle mutants and growth in different growth media. Besides, we have used this tool to uncover the role of the MAPK Sty1 on cell cycle, which has been classically linked to cell cycle due to its elongated phenotype. Existing studies linking Sty1 to cell cycle regulation have primarily relied on genetic interactions with *cdc25* [13], measuring cell length at septation [14] or through experiments of block-and-release using temperature sensitive strains which might have a negative effect by themselves on cell cycle [15]. Using this system to study the role of Sty1, we observed that the kinase has a minimal, if any, direct impact on cell cycle progression. This finding is significant because Sty1 has long been implicated in the control of cell cycle checkpoints under both unperturbed and stress conditions. Our data, however, suggest that the elongated cell phenotype observed in *sty1*-deficient cells is not due to any substantial change in the cell cycle itself but rather to an increase in the cell growth rate. This suggests that the role of Sty1 role may be more related to regulating cellular growth than directly modulating the timing of cell cycle transitions. Overall, our novel cell cycle reporter system provides a powerful tool for studying cell cycle dynamics at the single-cell level.

## Results

### Genetically encoded cell cycle reporter

**Fig 1:**
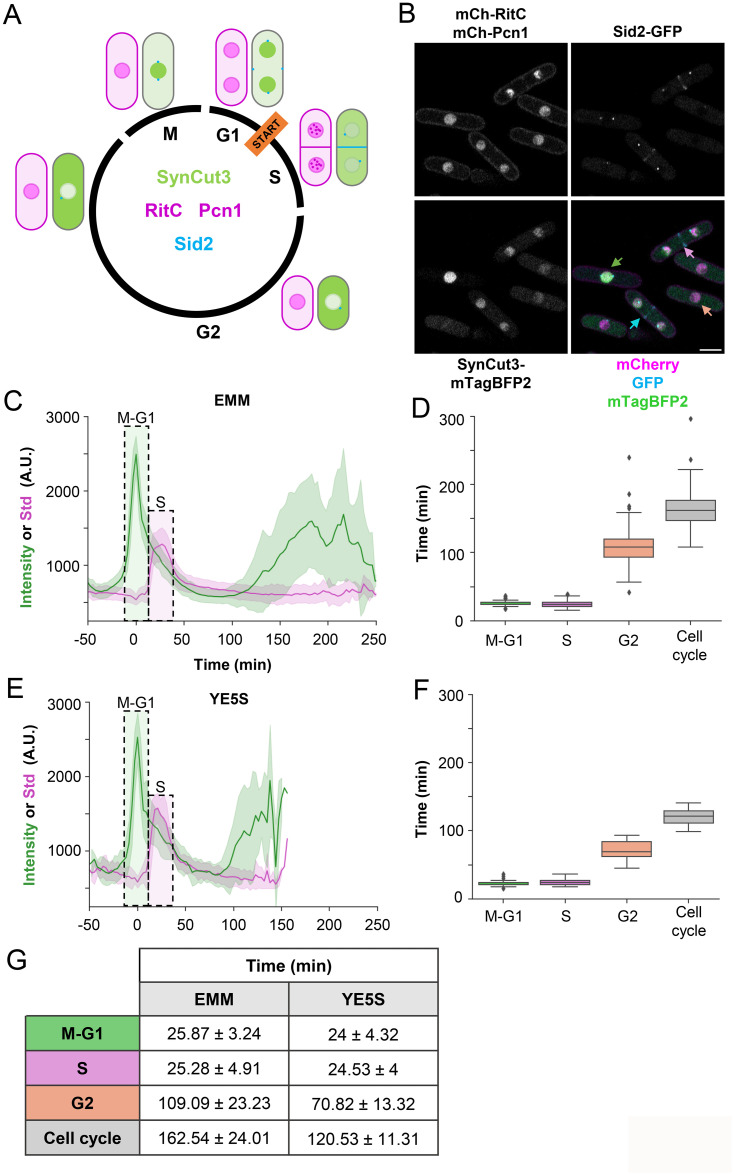
Cell cycle reporter strain, FLCCR. (A) Scheme depicting all the fluorescent markers and their localization along the cycle: Pcn1 (magenta, nuclear), RITC (magenta, membrane), Sid2 (cyan, spindle pole body and septum), SynCut3 (green, nuclear in mitosis, cytoplasmic in S-G2). (B) Airyscan microscopy of FLCCR strain. Arrows in merged image indicate cells in G1 (cyan), S (magenta), G2 (salmon), or mitosis (green). Scale bar = 5 μm. (C, E) Nuclear SynCut3 mean fluorescence and Pcn1 Std fluorescence of cells grown in EMM (C) or YE5S (E). Individual cycles were synchronized to peak SynCut3 nuclear intensity. Green box marks M-G1 phases, magenta box marks S phase. (D, F) Quantification of the different cell cycle phases of cells grown in EMM (D) or YE5S (F). Boxplot represents quartile distribution. Outliers are depicted as dots. (G) Table shows mean ± standard deviation. EMM *n* = 224 cells; YE5S *n* = 37 cells.

Traditionally, assessing the timing of cell duplication in single-celled organisms has relied on monitoring the optical density of a culture over a restricted timeframe. However, this method overlooks variations in cell size across different strains. Alternatively, the use of a Neubauer chamber provides snapshots at various culture times but fails to differentiate between live and dead cells. Given the limitations of these traditional methods, we developed a reporter system, FLCCR, capable of not only measuring the duration of the entire cell cycle but also discerning the duration of each phase in time-lapse experiments. This reporter system comprises 4 distinct fluorescent markers: SynCut3 (which localizes to the nuclei during mitosis) [16], Pcn1 (which exhibits a punctate pattern during S phase), Sid2 (marking the spindle pole body and the cell division site), and RitC (labeling the cellular membrane) [17]. The combination of these markers enables precise identification of each cell cycle phase and determination of the total cycle duration (Fig 1A). Importantly, the inclusion of these markers does not compromise cell viability or growth compared to an isogenic strain lacking the markers (S1AB Fig).

Next, we quantified the different cell cycle phases in asynchronous cultures through time-lapse experiments (Fig 1B and S1 Movie) or snapshots from the same cultures (S1C Fig). In our time-lapse experiments, individual cells were tracked, with the peak nuclear fluorescence of SynCut3 indicating mitosis and the maximum standard deviation of Pcn1 fluorescence signifying the peak of S phase (Fig 1C). While the extremely short G1 phase in fission yeast precludes resolution in our 3-min interval experiments, we determined a wild-type strain’s cell cycle length to be 165.26 ± 26.58 min, with M+G1 accounting for 16% and S phase for 14% of the cycle duration (Fig 1D and 1G). When cells were grown in rich media (YE5S), we observed a shortening of the cell cycle duration, mainly because the G2 phase was reduced (Fig 1E–1G). To differentiate M from G1, we repeated the time-lapse experiments, capturing images every minute within a 3-h window to avoid reporter signal photobleaching (S1D Fig). Under these conditions, we estimated G1 duration (time between the maximum separation of nuclei and entry into S phase) in a wild-type strain as 3.5 ± 1.65 min (S1E and S1F Fig).

### Validation of the reporter in different cell cycle mutants

**Fig 2:**
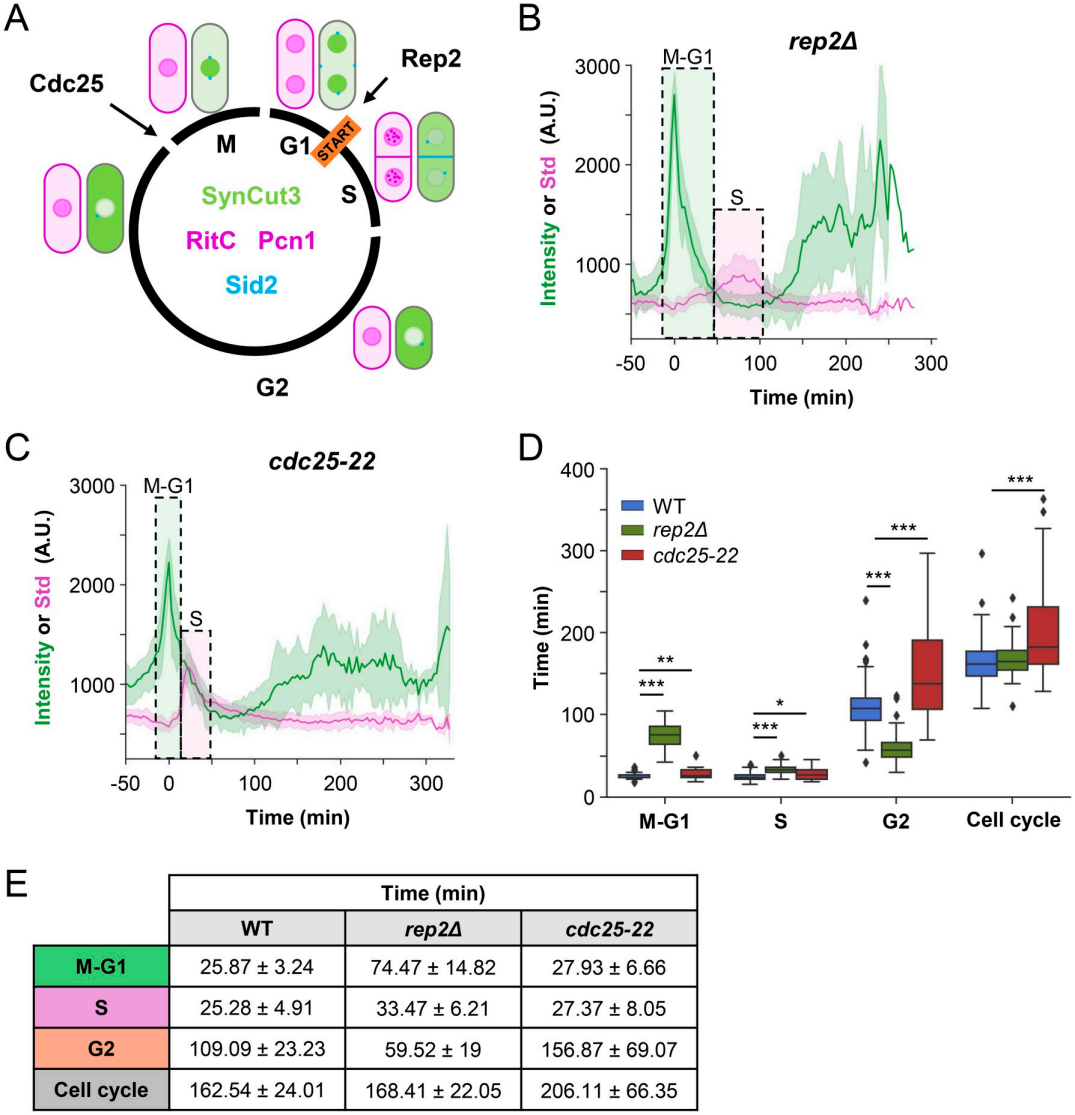
FLCCR validation. (A) Scheme of the cell cycle indicating the main point of action of Rep2 and Cdc25. Rep2 promotes START by MBF activation and Cdc25 promotes mitosis entry by activating the CDK Cdc2. (B, C) Nuclear SynCut3 mean fluorescence and Pcn1 Std fluorescence in a *rep2Δ* strain (B, *n* = 51) and in a *cdc25-22* strain grown at 30°C (C, *n* = 34). (D, E) Quantification of the different cell cycle phases. Boxplot represents quartile distribution. Outliers are depicted as dots. Table shows mean ± standard deviation. WT *n* = 224 cells, *rep2Δ n* = 51 cells, and *cdc25-22 n* = 34 cells. *: *p* < 0.05; **: *p* < 0.01; ***: *p* < 0.001.

Next, we decided to test FLCCR in conditions in which either G1/S or G2 phases were extended. For extended G1/S, we utilized a *rep2Δ* strain known for slow S phase progression and G1 arrest at low temperatures [18] (Fig 2A). Conversely, to induce an extended G2 phase, we employed a strain harboring a *cdc25* allele (*cdc25-22*) that exhibits delayed G2/M transition at the semi-permissive temperature of 30°C [19] (Fig 2A). These mutations were introduced into the FLCCR strains bearing the 4 fluorescent markers, which did not compromise survival in spot assays or growth in liquid cultures (S2AB Fig).

Following the same experimental setup employed with the wild-type strain, subsequent analysis of asynchronous cultures through time-lapse experiments, with images captured every 3 min over a span of 10 h (S2C Fig), unveiled notable alterations in the cell cycle dynamics. In the *rep2Δ* strain (Fig 2B and S2 Movie), while the overall cell cycle length exhibited a marginal increase from 159.34 min in the wild type to 170.50 min, there was a notable extension of the M/G1 phase (72.93 min versus 26.67 min in the wild type) alongside a concomitant shortening of the G2 phase (62.16 min versus 104.21 min in the wild type). This reduction in G2 phase duration sufficiently compensated for the extended G1 phase, maintaining a normal cycle length (Fig 2D and 2E). Conversely, analysis of the *cdc25-22* strain showed a cell cycle length prolonged by 43.57 min exclusively attributable to an extended G2 phase (156.87 min versus 107.23 min in the wild type) (Fig 2C and S3 Movie). Given the inherently brief durations of G1 and S phases in fission yeast, no compensatory mechanism was feasible to counteract the lengthening of the cell cycle in the *cdc25-22* background if there was any kind of cell size homeostasis (Fig 2D and 2E).

We assessed the performance of the FLCCR system in genetic backgrounds known to potentially influence the duration of the G2 phase, resulting in cells longer than wild-type cells. Our selection of mutants included *cdr1Δ* and *cdr2Δ*, implicated in the mitotic G2 cell size control checkpoint signaling [20], alongside a strain carrying 5 copies of the *wee1* gene (*5xwee1*), inducing a G2/M transition delay [21]. While these 3 strains exhibited larger cell sizes compared to the wild-type strain, the cell cycle remained unaffected in *cdr1Δ* and *cdr2Δ* strains, with only minor impact observed in the *5xwee1* strain (S2D Fig).

### Integrating the cell cycle reporter with active growth measurement at cell tips for quantitative cell size prediction

We postulated that the distinct growth rates among the tested strains might account for the observed differences in the final cell size. To explore this hypothesis, we determined the growth rates in these strains and the wild type by measuring size differences across 10 time points (equivalent to 30 min) after septation and before entry into mitosis. Remarkably, the growth rates obtained under these conditions in a wild-type background closely mirrored those previously obtained using 2 different fluorescent markers [10]. Notably, while *rep2Δ* exhibited a growth rate similar to the wild type, the remaining strains displayed heightened growth rates (ranging from 30% to 45% increase; see below), which in itself could justify their larger size without impacting the length of the cell cycle.

**Fig 3:**
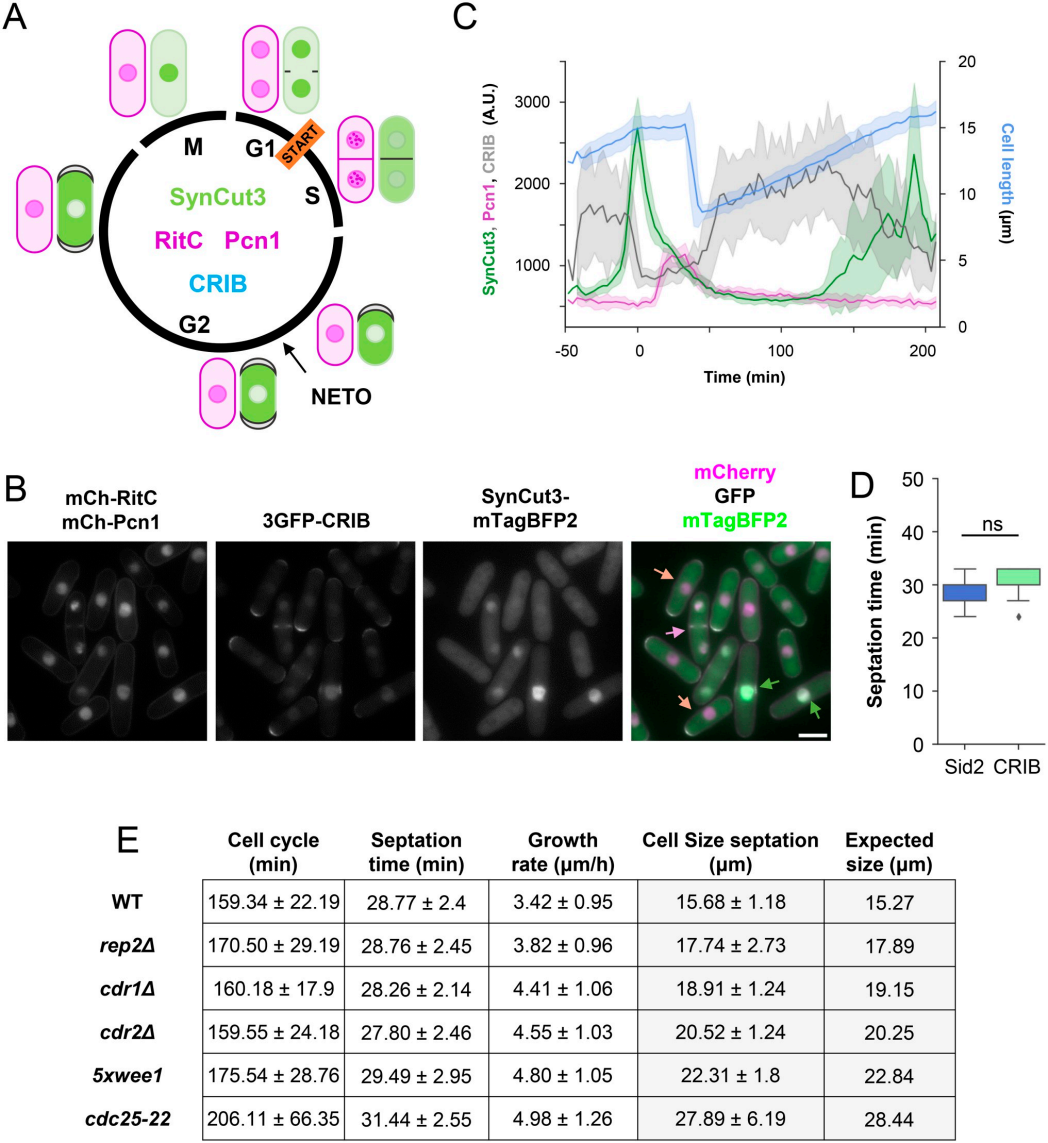
Measurement of different growth parameters. (A) Scheme depicting all the fluorescent markers and their status along the cell cycle. Pcn1 (magenta, nuclear), RITC (magenta, membrane), CRIB (gray, growing tips and septum), SynCut3 (green, nuclear in mitosis, cytoplasmic in S-G2). (B) Epifluorescence microscopy of the CRIB and FLCCR strain. Arrows in merged image signal S phase (magenta), G2 (salmon) and mitosis (green). Scale bar = 5 μm. (C) Signal profile of nuclear SynCut3-mTagBFP2 (green), nuclear mCherry-Pcn1 (magenta), CRIB-3GFP at old-growth tips (gray), and cell length (blue) during the cell cycle. Individual cycles were synchronized to peak SynCut3 nuclear intensity. *n* = 17 cells. (D) Quantification of the septation time (time from Sid2 appearing at the septum until cell separation by RitC) using Sid2 or CRIB localization at the septum as septation initiation mark. Cytokinesis end was considered when a parental cell splits into 2 daughter cells. Sid2 *n* = 49 cells, CRIB *n* = 54 cells. (E) Table showing the mean ± SD of all the growth parameters that can be measured with the cell cycle reporter strain. Different cell cycle mutants were used to determine the measured and the calculated cell size (*rep2Δ* for G1/S; *cdr1Δ*, *cdr2Δ*, *5xwee1* and *cdc25-22* for G2/M).

However, a consideration arose in this calculation: the growth rate is not uniform during the cell cycle. During septation, for instance, cells cease growth at their tips. This phenomenon is readily observable using an active growth marker like CRIB [10]. To precisely quantify the duration of halted growth, we incorporated GFP-CRIB into the FLCCR system, omitting Sid2-GFP, which also labels the septum (Fig 3A). The strain with this modification enabled us to gauge CRIB intensity at cell tips across the different cell cycle phases (Fig 3B and 3C and S4 Movie) and ascertain that the period that cells spend in septation corresponds with the cessation of cell growth (Figs 3D and S3). Then, we integrated this information into our cell size prediction model as follows:

$$\begin{array}{c} \text{Growth}=\text{Growth}\;\text{rate}\;\text{x}\left(\text{Cell}\;\text{cycle}\;\text{duration}–\text{Septation}\;\text{Time}\right)\\ \text{Final}\;\text{Cell}\;\text{Size}=\text{Newborn}\;\text{size}+\text{Growth} \end{array}$$

With this straightforward calculation, we were able to determine that the expected size of a wild-type cell was 15.17 μm, which is remarkably close to the actual measurement of 15.68 μm (Fig 3E). To validate our approach, we applied the same calculation to other mutant strains used in our study and found that our method reliably predicts cell size, with a maximum error of less than 3% (wild type and *5xwee1*).

### Normal cell cycle length and distribution in Sty1-deficient cells

**Fig 4:**
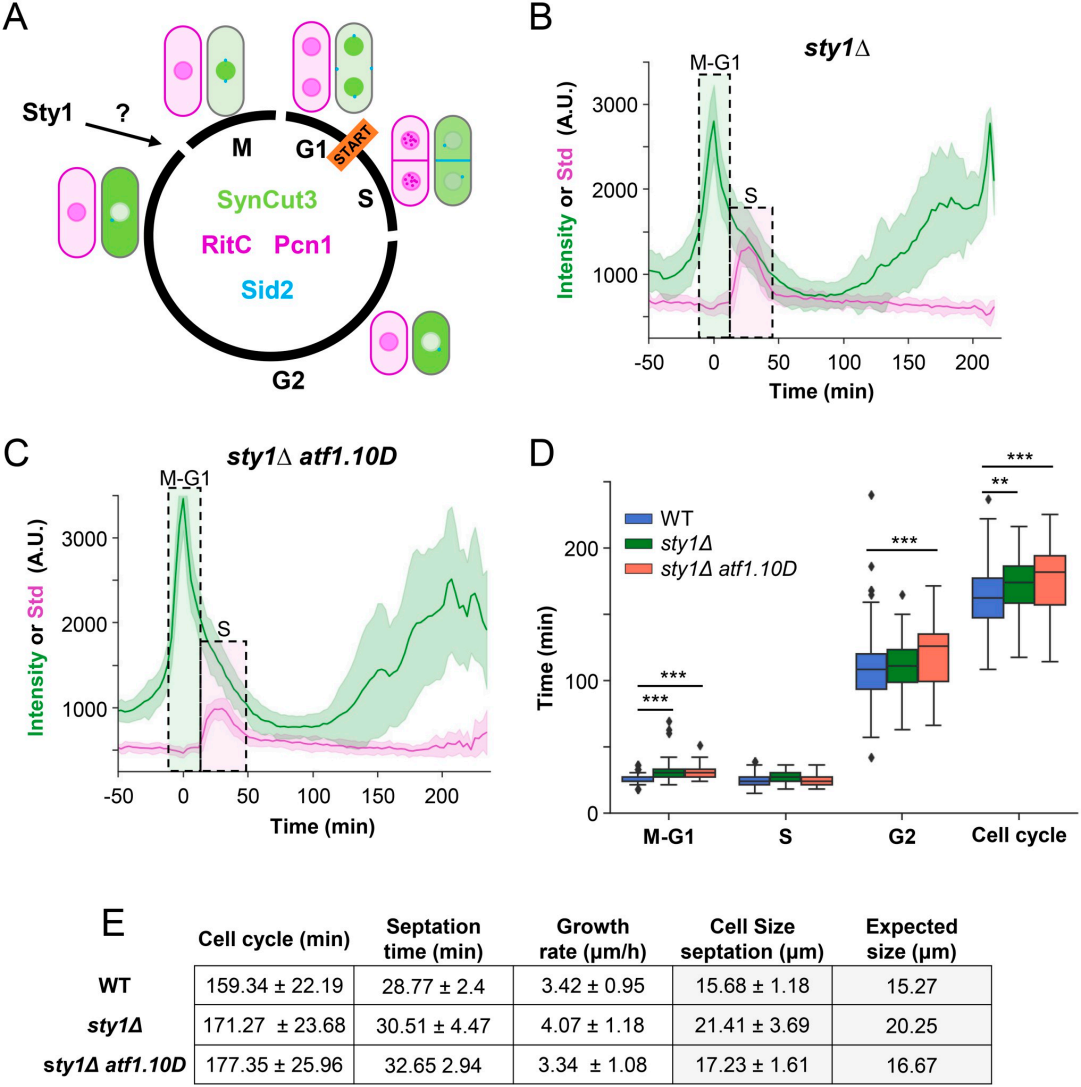
Cell cycle measurement in Sty1 mutants. (A) Scheme depicting all the fluorescent markers and their localization along the cycle: Pcn1 (magenta, nuclear), RITC (magenta, membrane), Sid2 (cyan, spindle pole body and septum), SynCut3 (green, nuclear in mitosis, cytoplasmic in S-G2). The arrow indicates the putative point of regulation of the cell cycle by Sty1. (B, C) Nuclear SynCut3 mean fluorescence and Pcn1 Std fluorescence of a *sty1Δ* (B) and *sty1Δ atf1*.*10D* (C) strains. (D) Boxplot represents quartile distribution. Outliers are depicted as dots. (E) Mean and SD of the quantification of all growth parameters measured with the FLCCR. WT *n* = 224 cells, *sty1Δ n* = 43 cells, and *sty1Δ atf1*.*10D n* = 65 cells. *: *p* < 0.05; **: *p* < 0.01; ***: *p* < 0.001.

The MAP kinase Sty1, on top of being essential for the cells to survive stress situations, has been believed for more than 30 years to be involved in the regulation of cells cycle under unstressed conditions [13,14]. To elucidate the precise role of Sty1 in cell cycle regulation, extending beyond the established genetic interactions with the Cdc25 phosphatase coding gene (Fig 4A), we deleted *sty1* in the FLCCR strain. Additionally, as a control, we deleted *sty1* in a strain expressing a constitutively active Atf1 (*atf1*.*10D*), which mimics the Sty1-dependent phosphorylations, thereby generating an Atf1 variant whose activity remains independent of Sty1 [22]. When either of these 2 mutations was introduced into the FLCCR strain containing the 4 fluorescent markers, the resulting strains behaved similarly to the original strains indicating the fluorescent labeled proteins in the FLCCR strain did not alter the phenotype of *sty1Δ* or *sty1Δatf1*.*10D* (S4A and S4B Fig). Using the same experimental setup as with the wild-type strain, analysis of asynchronous cultures through time-lapse experiments, capturing images every 3 min over a period of 10 h (Figs 4B–4D and S4C and S5 and S6 Movies), revealed no major changes in the length of the cell cycle or in the distribution of the different phases. In any case, the strain that exhibited the most significant deviation from the wild-type strain was the *sty1Δ atf1*.*10D* mutant, which is supposed not to have affected its cell cycle progression (Figs 4E and S4D). Although *sty1Δ* cells display an almost unchanged cell cycle length and an unperturbed distribution of the phases, they exhibit a significantly larger size compared to wild-type cells (21.41 μm versus 15.68 μm of wild type). This observed increase in size aligns with our predicted size calculation of 20.25 μm (Fig 4F).

**Fig 5:**
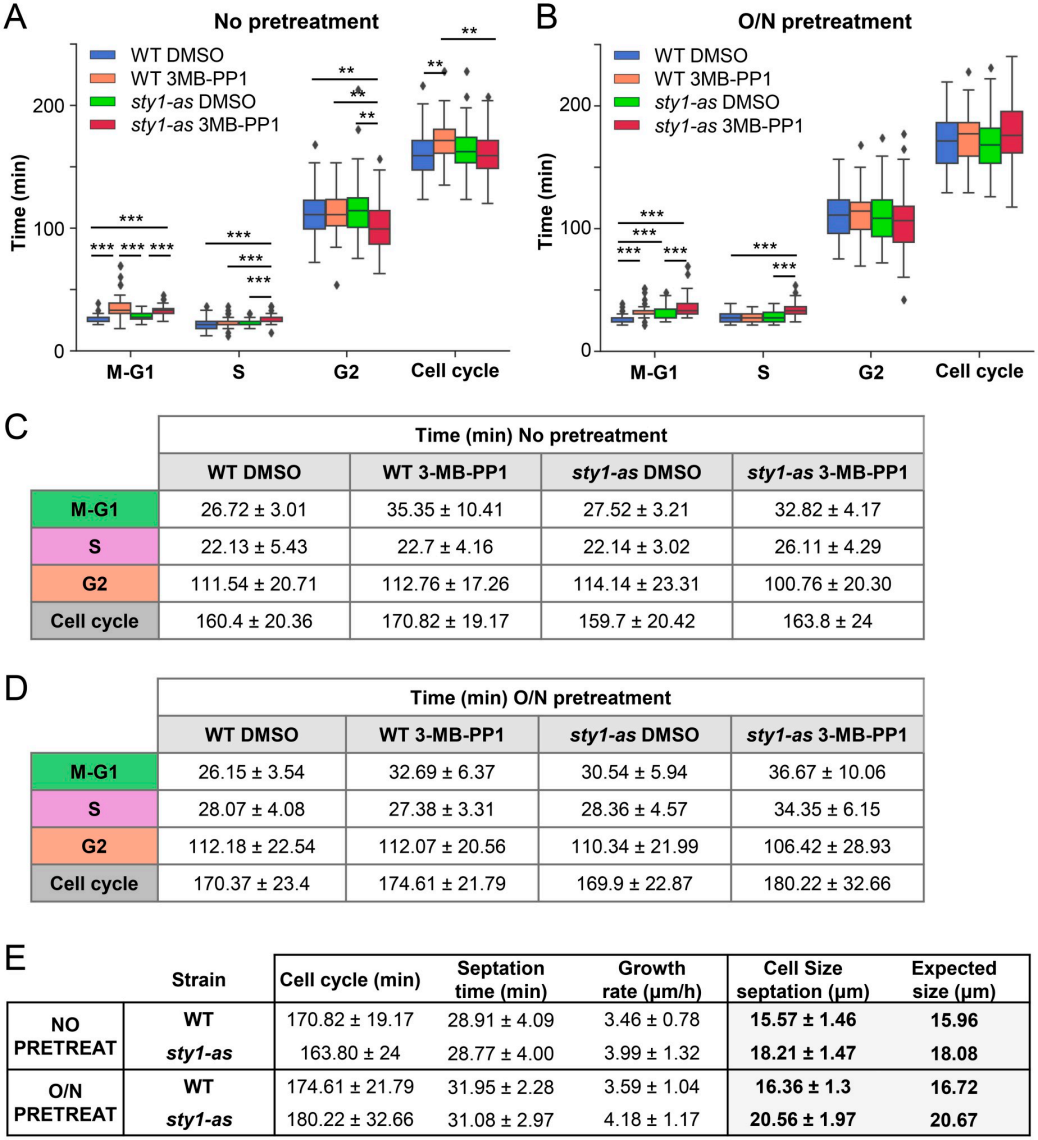
Sty1 chemical inhibition does not alter cell cycle progression. (A, C) Quantification of the different cell cycle phases of WT and *sty1-as* without pretreatment. Boxplots (A) represent quartile distribution with outliers depicted as dots and table (C) shows mean ± standard deviation. WT DMSO *n* = 66 cells, WT 3MB-PP1 *n* = 51 cells, *sty1-as* DMSO *n* = 64 cells, *sty1-as* 3MB-PP1 *n* = 51 cells. *: *p* < 0.05; **: *p* < 0.01; ***: *p* < 0.001. (B, D) Quantification of the different cell cycle phases of WT and *sty1-as* after an overnight pretreatment with 10 μm 3MB-PP1. Boxplots (B) represent quartile distribution with outliers depicted as dots and table (D) shows mean ± standard deviation. WT DMSO *n* = 53 cells, WT 3MB-PP1 *n* = 40 cells, *sty1-as* DMSO *n* = 55 cells, *sty1-as* 3MB-PP1 *n* = 40 cells. *: *p* < 0.05; **: *p* < 0.01; ***: *p* < 0.001. (E) Mean and SD of the quantification of all growth parameters measured with the FLCCR.

However, there was a caveat using a constitutively Sty1-deficient strain, given the pleiotropic phenotypes of this strain, ranging from heightened sensitivity to various extracellular and intracellular stresses to a shortened lifespan [23,24]. To circumvent potential issues arising from long-term adaptation in a Sty1-deficient strain, we decided to use a conditional allele of *sty1*. We used a *sty1-as* allele that allowed the inhibition of Sty1 with a bulky analog of ATP [24,25]. The growth of all these strains remained unaffected under unstressed conditions, whether in a wild-type background or when incorporated into the FLCCR background (S5A and S5B Fig). We validated the efficacy of 3-MB-PP1 treatment by confirming the lack of Atf1 phosphorylation upon H_2_O_2_ stress (S5C Fig). To assess the impact of conditional Sty1 inactivation, we analyzed asynchronous cultures of wild-type and *sty1-as* strains treated with 3-MB-PP1. In one set of experiments, 3-MB-PP1 was administered just before initiating image capture every 3 min (Fig 5A and S7–S10 Movies), while in another set, cells were pre-treated with 3-MB-PP1 for 12 h before imaging (Fig 5B and S11–S14 Movies). Surprisingly, as depicted in Fig 5C and 5D, no significant alterations were observed in the cell cycle length or phase distribution upon conditional Sty1 inactivation compared to wild-type cells. However, we noted a proportional increase in cell size corresponding to the duration of Sty1 inactivation with 3-MB-PP1 (Fig 5E). This observed increase in cell size appears to be solely sustained by an elevated cell growth rate (Fig 5E).

Another approach to discern the possible effect of Sty1 on cell cycle regulation was to investigate previously described genetic interactions. Since the first one described was with *cdc25*, we generated a double mutant, *cdc25-22 sty1-as*. Upon Sty1 inhibition with the ATP analog, cells acquired the elongated phenotype typical of *sty1Δ* cells, which was even more pronounced in the *cdc25-22 sty1-as* double mutant (S5D Fig). Despite this effect, we observed no significant changes in the distribution or duration of the various cell cycle phases (S5E Fig). Interestingly, when cultures were left to reach saturation and cells entered the stationary phase, *sty1-as* cells became smaller, while *cdc25-22 sty1-as* cells grew even larger. A second genetic interaction reported in the literature involves the phosphorylation of Polo kinase Plo1 at Ser402 by Sty1, either directly or indirectly. It was previously shown that a phosphomimetic mutation *plo1*.*S402E* can partially rescue the *cdc* phenotype in *sty1Δ* cells. However, when we introduced this same mutation into the *sty1-as* strain, we did not observe the expected effect on cell size (S5F Fig), suggesting that the genetic interaction between Sty1 and Plo1 does not depend on Sty1 kinase activity.

In summary, we have engineered a reporter strain, FLCCR, enabling single-cell cycle analysis. Using FLCCR, we have shown that under unstressed conditions, the MAP Kinase Sty1 exerts no significant influence on cell cycle progression. Moreover, we attribute the larger size of cells lacking functional Sty1 to an elevated polarized growth rate.

## Discussion

This study provides the use of a tool that allows the quantification of the duration of the different phases of the cell cycle in fission yeast, allowing the determination of the length of the cell cycle, cell length, or the growth rate, respectively. Using FLCCR, we have been able to determine that several mutations that render elongated cells do not necessarily have a problem with the control of the cell cycle core, since their longer phenotype can be simply explained by an increased cell growth rate. Among these, the MAP kinase Sty1 is the archetype that has always been believed to be regulating cell cycle progression, even in the absence of an exogenous stress. Here, we show that Sty1 has, in fact, a minimal impact on unaltered cell cycle progression and that its large cell size phenotype can be almost exclusively explained by their increased cell growth rate.

The development of genetically encoded cell cycle reporters was a significant advancement in single-cell cycle analysis, offering an improved understanding of the intricacies underlying cell division dynamics. The Fluorescent Ubiquitination-based Cell Cycle Indicator (FUCCI) system, developed by Miyawaki and colleagues, revolutionized the study of cell cycle dynamics in live metazoans cells [26]. This innovative tool used 2 fluorescent probes, Fucci-G1 and Fucci-S/G2/M, which undergo cell cycle-dependent changes in expression levels, allowing for the visualization and tracking of cells in different phases of the cell cycle based on their fluorescence. While the FUCCI system offered numerous advantages for studying cell cycle dynamics, it also had some limitations that should be considered. One limitation is related to the specificity of the probes used in the FUCCI system. The Fucci-G1 probe, which marks cells in the G1 phase, relies on the degradation of the Cdt1 protein. Similarly, the Fucci-S/G2/M probe, which marks cells in the S, G2, and M phases, is based on the degradation of the Geminin protein. However, the degradation of Cdt1 or Geminin can be influenced by factors other than cell cycle progression, such as DNA damage, stress responses, changes in protein stability or expression levels, and any of them could potentially affect the accuracy of cell cycle phase identification. Additionally, the FUCCI system requires the expression of exogenous fluorescent proteins, which could potentially interfere with normal cellular processes. High expression levels of fluorescent proteins may impose metabolic burdens on cells or perturb endogenous signaling pathways, leading to unintended effects on cell behavior.

Despite these limitations, the FUCCI system has remained so far a valuable tool for studying cell cycle dynamics in live metazoan cells. We have shown that our cell cycle reporter, FLCCR, is a robust system that can overcome some of the limitations of FUCCI. So far, we have shown that cells expressing the 4 fluorescent markers, behave exactly as their progenitors without the fluorescent proteins. And even in the case of cells that present some challenge during the DNA synthesis (like *rep2Δ*), our system allows the correct positioning of any given cell in a specific moment of the cell cycle.

Several aspects of this study could be further refined to enhance its robustness and applicability. While it is feasible to conduct time-lapse experiments capturing images every minute, this approach often leads to photobleaching, significantly shortening the duration of the experiment. Consequently, this limitation impedes the ability to track a sufficient number of cells through an entire cell cycle accurately. Balancing image acquisition frequency with photobleaching mitigation strategies could optimize experimental conditions, allowing for more comprehensive cell cycle monitoring. On the other hand, the behavior of the FLCCR strain under conditions where cells deviate from normal cell cycle progression remains unclear. Specifically, understanding how these reporters respond when cells enter the stationary phase or when they re-enter the cell cycle is essential for elucidating their dynamics beyond standard growth conditions. Future investigations exploring the stability of the FLCCR system under various physiological states will provide valuable insights into its reliability and versatility across different contexts.

## Materials and methods

### Yeast strains, plasmids, and growth conditions

All *S*. *pombe* strains are listed in S1 Table and were grown in EMM or YE5S as described previously [27]. The cell cycle reporter strain was constructed in the following way: Sid2-GFP strain was obtained from elsewhere [28]. All the plasmids used in this work are listed in S2 Table. pAV plasmids [17] were obtained from the National Biosource Project (NBRP). In order to stably integrate the mCherry-Pcn1 tagging into the *lys3* locus, we isolated a 5.7 kb region from pAV0785 [25] containing the *pcn1* promoter, followed by mCherry-Pcn1, the *nmt1* terminator and part of the TEV promoter using KpnI. The insert was cloned into pAV0357 [17], yielding pAY1035. synCut3 plasmid was kindly given by Paul Nurse [9]. mTagBFP2 was extracted from pAV0816 and used to replace the original mCherry from SynCut3-mCherry plasmid and generate SynCut3-mTagBFP2 plasmid pAY1193. Pcn1, RitC, and SynCut3 were generated by stable integration of plasmids pAV0607, pAY1035 and pAY1193. Gene deletions were performed using a polymerase chain reaction (PCR)-based method and were confirmed by PCR on genomic DNA. To generate the *cdc25-22 (cdc25-C532Y)* and the *sty1-as* (*sty1-T97A*) and *plo1-S402E* reporter strains, *SpEDIT* CRISPR/Cas9 method was used [29] using the primers listed in S3 Table.

### Growth curves

Growth curves were performed as previously described [30]. Briefly, exponentially growing cultures were diluted to an initial OD_600_ = 0.1 and were inoculated per duplicate in a 96-well non-coated polystyrene microplates with an adhesive seal. Plates were incubated in a Power Wave microplate scanning spectrophotometer (Bio-Tek) at 30°C with continuous shaking. OD_600_ was automatically recorded every 10 min for 48 h using Gen5 software. Graphs were made using Pandas, Pyplot, and Seaborn packages from Python.

### Spot survival assays

Assays were performed as previously described [31]. Cells were grown at 30°C in EMM until they reached logarithmic phase to an OD_600_ = 0.5. The same number of cells (10^5^ and 1/10 serial dilutions) in 3 μl were spotted on plates that also contained 10 μm 3MB-PP1, H_2_O_2_, or HU where indicated. The spots were allowed to dry and the plates were incubated at 30°C (or at the indicated temperature) for 2 to 4 days.

### TCA extracts and immunoblot analysis

To analyze Atf1 phosphorylation, cells were grown in EMM to log phase (OD_600_ = 0.5). Sty1-as inhibition was done using 10 μm 3MB-PP1 (A602960, Toronto Research Chemicals) 15 min prior 1 mM H_2_O_2_ treatment for 5 min. Protein extraction was performed as previously described [31]. Trichloroacetic acid (TCA) was added to a final concentration of 10%. Cells were pelleted and washed in 20% TCA. The pellets were resuspended in 100 μl of 12.5% TCA and lysed by vortexing in the presence of glass beads. Cell lysates were pelleted, washed in 1 ml ice-cold acetone, and dried. Pellets were resuspended in Tris buffer (0.1 M Tris—HCl (pH 8.0), 1 mM EDTA, 1% SDS), loading buffer was added and samples were boiled for 5 min at 100°C. Samples were separated by SDS-PAGE and detected by immunoblotting with polyclonal anti-Atf1 [31].

### Epifluorescence microscopy imaging

Cells were grown in filtered EMM (or in YE5S, when indicated) to logarithmic phase (OD_600_ = 0.5) and were imaged on a Nikon Eclipse 90i microscope equipped with differential interference contrast optics, a PLAN APOVC100x 1.4 oil immersion objective, an ORCA-II-ERG camera (Hamamatsu), excitation and emission filters UV, GFP4050B and mCherry-C (Semrock, West Henrietta, New York, USA), image acquisition software Metamorph 7.8.13 (Gataca Systems), and a LED illumination Cool LED pE-300lite.

### Life-cell airyscan imaging and time-lapse experiments

Cells were grown in filtered EMM until reaching logarithmic phase OD600 = 0.5. Then, 0.2 ml of the culture was placed in a well from 8-chamber Ibidi #1.5 Borosilicate Coverglass System (Ibidi 80827) previously coated with 40 μl of 1 mg/ml soybean lectin (L1395, Sigma-Aldrich). Cells were left 1 min to attach to the bottom and then medium was carefully removed, washed with 0.2 ml of previously warmed media and finally left in 0.2 ml of fresh tempered medium. Then, cells were quickly placed in the microscopy chamber at 30°C.

Image acquisition was done with an emission scanning spectral confocal microscope Zeiss-LSM-980 with Airyscan 2, using a 63× Plan-APO 1.46 NA objective. A 2× digital zoom was used to reach Nyquist sampling. Excitation lasers used were 405 nm for mTagBFP2, 488 nm for GFP, and 561 nm for mCherry. Filters used were a double BP 420–475 + 500–545 for GFP and a double BP 420–480 + 570–630 for mTagBFP2 and mCherry. Image acquisition software used was ZEN 3.6. A z-stack of 3 μm in 5 slices was recorded during 10 h per position with a frequency of 3 min using the Airy 8Y mode. After acquisition, time-lapse files were Airyscan processed with standard values and a maximum z-projection was performed to collapse all z-positions.

### Time-lapse image analysis

Image analysis was performed using the TrackMate plugin [32] from Image J [33]. A Laplacian of Gaussian (LoG) particle detector was used on red channel and particle diameter was set at 2.2 μm or 3.0 μm depending on nuclei size. An automatic first quality threshold was applied without a second filtering. To follow nuclei during the time-lapse, a simple LAP tracker algorithm was used with a maximum linking distance of 5 μm, a maximum gap-closing distance of 5 μm and a maximum frame gap-closing of 10 frames. Then, the numerical values were extracted as a.csv file and manually analyzed using excel. Mean intensity values were used to follow SynCut3-mTagBFP2 and standard deviation of intensity values were used to follow DNA synthesis in mCherry-Pcn1. Individual cell cycles were synchronized to maximum SynCut3-mTagBFP2 values. To distinguish between the different cell cycle phases, an increase of 100% SynCut3 nuclear signal from baseline was used as threshold determination for mitosis entry, and an increase of 50% of mCherry-Pcn1 Standard Deviation from baseline was used as threshold for S-phase delimitation. Graphs were made using Pandas, Pyplot, and Seaborn packages from Python.

Cell size at septation was measured from cell pole to cell pole using the straight-line tool from Fiji when Sid2-GFP appeared at the septum. Septating time was measured as the time lapse between Sid2-GFP or GFP-CRIB fluorescence appeared at the septum and the 2 daughter cells were clearly separated. Growth rate was measured using the straight-line tool from Fiji when cells were under growth time (considered as non-septating time). CRIB signal at cell tips was measured as described in [17]. Briefly, the rectangle square from Fiji was used to measure the maximum CRIB signal, comprising only the cell tip from one cell.

## Supporting information

S1 Table List of yeast strains used in this work. (DOCX)

S2 Table List of plasmids used in this work. (DOCX)

S3 Table CRISPR oligonucleotides used in this work. (DOCX)

S1 Fig Characterization of FLCCR. (A) Serial dilutions of the WT and FLCCR strains in YE5S or EMM plates. All plates were grown at 30°C, unless indicated. H2O2 or hydroxyurea (HU) were added at the indicated concentrations. (B) Growth curves comparing growth at 30°C of a WT and FLCCR strain in YE5S or EMM media. (C) Epifluorescence microscopy of the FLCCR strain. Arrows in merged image indicate a cell in S phase (magenta), G2 phase (salmon) or mitosis (green). Scale bar = 5 μm. (D) Nuclear SynCut3 mean fluorescence and Pcn1 Std fluorescence. Individual cycles were synchronized to peak SynCut3 nuclear fluorescence. To distinguish G1 from mitosis, we considered that mitosis was finished when the cell achieved the maximum distance between the 2 nuclei and G1 was defined as the time between the maximum separation of nuclei and entry into S phase. Green box marks mitosis, blue box marks G1 and magenta box marks DNA synthesis. *n* = 23 cells. (E, F) Quantification of the duration of the different cell cycle phases. Boxplot represents quartile distribution. Outliers are depicted as dots. Table shows mean ± standard deviation. *n* = 23 cells.
(TIF)

S2 Fig Validation of FLCCR in backgrounds with extended G1 or G2 phases. (A) Serial dilutions of the WT, *rep2Δ* and *cdc25-22* strains in WT or FLCCR background. Plates were grown at 30°C for 3 days in minimal medium (MM). (B) Growth curves comparing growth at 30°C of a WT, *rep2Δ* and a *cdc25-22* in WT or FLCCR backgrounds. *n* = 3 experiments. (C) Airyscan microscopy of a *rep2Δ* (top row) or a cdc25-22 (bottom row) in the cell cycle reporter background. Scale bar = 5 μm. (D) Quantification of the duration of the different cell cycle phases, shown as mean ± standard deviation. WT *n* = 224 cells; *cdr1Δ n* = 48 cells; *cdr2Δ n* = 58 cells; *5xwee1 n* = 35 cells.
(TIF)

S3 Fig Comparison of the cell cycle reporter strain using Sid2 or CRIB as a mark for septation of tip growth. (A) Mitosis in a Sid2 FLCCR background. Time lapse between images is 3 min. Yellow arrow marks septation initiation and red arrow marks end of cytokinesis. Scale bar = 5 μm. (B) Mitosis in a CRIB combined with FLCCR. Time lapse between images is 3 min. Yellow arrow marks septation initiation and red arrow marks end of cytokinesis. Scale bar = 5 μm.
(TIF)

S4 Fig Cell cycle is unaffected in Sty1 depleted cells. (A) Serial dilutions of the WT, *sty1Δ* and a *sty1Δ atf1*.*10D* strains in WT or FLCCR background. Plates were grown at 30°C for 3 days in minimal medium (MM). (B) Growth curves comparing growth at 30°C of a WT, *sty1Δ* and a *sty1Δ atf1*.*10D* in WT or FLCCR backgrounds. *n* = 4 experiments. (C) Airyscan microscopy of a *sty1Δ* and a *sty1Δ atf1*.*10D* in FLCCR background. Scale bar = 5 μm. (D) Quantification of the duration of the different cell cycle phases, shown as mean ± standard deviation. WT *n* = 224 cells; *sty1Δ n* = 43 cells; *sty1Δ atf1*.*10D n* = 65 cells.
(TIF)

S5 Fig Cell cycle is unaffected in sty1 conditional cells. (A) Serial dilutions of the WT, *sty1Δ* and a *sty1-as* strains in WT or FLCCR background. Plates were grown at 30°C for 3 days in minimal medium (MM), with DMSO or with 10 μm 3MB-PP1. (B) Growth curves comparing growth at 30°C of a WT (left) and *sty1-as* (right) in WT or in the cell cycle reporter backgrounds in the presence of DMSO or 10 μm 3MB-PP1. *n* = 3. (C) Western blot α-Atf1 to detect Atf1 phosphorylation under oxidative stress conditions. *sty1-as* strain was pre-treated with 10 μm 3MB-PP1 for 15 min before addition of 1 mM H_2_O_2_. *, unspecific band; upper arrow points to the phosphorylated form of Atf1; lower arrow points to non-phosphorylated form of Atf1. (D) Representative DIC microscopy images of FLCCR *sty1-as* and FLCCR *sty1-as cdc25-22* in logarithmic growth after 24 h of treatment with DMSO or 10 μm 3MB-PP1 or after 8 days in stationary phase with DMSO or 10 μm 3MB-PP1. Scale bar = 10 μm. (E) Table showing percentage (%) of cells in each cell cycle phase. FLCCR *sty1-as* (*n* = 187 cells) and FLCCR *sty1-as cdc25-22* (*n* = 179 cells). (F) Boxplot showing cell length at septation of *sty1-as* and *sty1-as plo1-S402E* with DMSO or 3MB-PP1 10 μm treatment after 24 h. The inset indicates the mean ± SD. *sty1-as* DMSO *n* = 32 cells, *sty1-as* 3MB-PP1 *n* = 44 cells, *sty1-as plo1-S402E* DMSO *n* = 51 cells, *sty1-as plo1-S402E* 3MB-PP1 *n* = 50 cells. *** = *p* < 0.001.
(TIF)

S1 Raw Image Raw image of S5C Fig. (TIF)

S1 Movie Growth of FLCCR WT cells in EMM. mCherry-Pcn1 (nuclear, magenta), mCherry-RitC (membrane, magenta), Sid2-GFP (cyan), and SynCut3-mTagBFP2 (green). Asynchronous growing cells at 30°C were imaged for 10 h with pictures taken every 3 min. Maximum-intensity projection of a 3 μm (0.75 μm step-size) is shown. Scale bar = 10 μm.
(AVI)

S2 Movie Growth of FLCCR *rep2Δ* cells in EMM. mCherry-Pcn1 (nuclear, magenta), mCherry-RitC (membrane, magenta), Sid2-GFP (cyan), and SynCut3-mTagBFP2 (green). Asynchronous growing cells at 30°C were imaged for 10 h with pictures taken every 3 min. Maximum-intensity projection of a 3 μm (0.75 μm step-size) is shown. Scale bar = 10 μm.
(AVI)

S3 Movie Growth of FLCCR *cdc25-22* cells in EMM. mCherry-Pcn1 (nuclear, magenta), mCherry-RitC (membrane, magenta), Sid2-GFP (cyan), and SynCut3-mTagBFP2 (green). Asynchronous growing cells at 30°C were imaged for 10 h with pictures taken every 3 min. Maximum-intensity projection of a 3 μm (0.75 μm step-size) is shown. Scale bar = 10 μm.
(AVI)

S4 Movie Growth of FLCCR CRIB cells in EMM. mCherry-Pcn1 (nuclear, magenta), mCherry-RitC (membrane, magenta), CRIB-3GFP (white), and SynCut3-mTagBFP2 (green). Asynchronous growing cells at 30°C were imaged for 10 h with pictures taken every 3 min. Maximum-intensity projection of a 3 μm (0.75 μm step-size) is shown. Scale bar = 10 μm.
(AVI)

S5 Movie Growth of FLCCR *sty1Δ* cells in EMM. mCherry-Pcn1 (nuclear, magenta), mCherry-RitC (membrane, magenta), Sid2-GFP (cyan), and SynCut3-mTagBFP2 (green). Asynchronous growing cells at 30°C were imaged for 10 h with pictures taken every 3 min. Maximum-intensity projection of a 3 μm (0.75 μm step-size) is shown. Scale bar = 10 μm.
(AVI)

S6 Movie Growth of FLCCR *sty1Δ atf1-10D* cells in EMM. mCherry-Pcn1 (nuclear, magenta), mCherry-RitC (membrane, magenta), Sid2-GFP (cyan), and SynCut3-mTagBFP2 (green). Asynchronous growing cells at 30°C were imaged for 10 h with pictures taken every 3 min. Maximum-intensity projection of a 3 μm (0.75 μm step-size) is shown. Scale bar = 10 μm.
(AVI)

S7 Movie Growth of FLCCR + DMSO cells in EMM. mCherry-Pcn1 (nuclear, magenta), mCherry-RitC (membrane, magenta), Sid2-GFP (cyan), and SynCut3-mTagBFP2 (green). Asynchronous growing cells at 30°C were imaged for 10 h with pictures taken every 3 min. DMSO treatment was added in the growth media used for imaging. Maximum-intensity projection of a 3 μm (0.75 μm step-size) is shown. Scale bar = 10 μm.
(AVI)

S8 Movie Growth of FLCCR + 10 μm 3-MB-PP1 cells in EMM. mCherry-Pcn1 (nuclear, magenta), mCherry-RitC (membrane, magenta), Sid2-GFP (cyan), and SynCut3-mTagBFP2 (green). Asynchronous growing cells at 30°C were imaged for 10 h with pictures taken every 3 min, and 10 μm 3-MB-PP1 treatment was added in the growth media used for imaging. Maximum-intensity projection of a 3 μm (0.75 μm step-size) is shown. Scale bar = 10 μm.
(AVI)

S9 Movie Growth of FLCCR *sty1-as* + DMSO cells in EMM. mCherry-Pcn1 (nuclear, magenta), mCherry-RitC (membrane, magenta), Sid2-GFP (cyan), and SynCut3-mTagBFP2 (green). Asynchronous growing cells at 30°C were imaged for 10 h with pictures taken every 3 min. DMSO treatment was added in the growth media used for imaging. Maximum-intensity projection of a 3 μm (0.75 μm step-size) is shown. Scale bar = 10 μm.
(AVI)

S10 Movie Growth of FLCCR *sty1-as* + 10 μm 3-MB-PP1 cells in EMM. mCherry-Pcn1 (nuclear, magenta), mCherry-RitC (membrane, magenta), Sid2-GFP (cyan), and SynCut3-mTagBFP2 (green). Asynchronous growing cells at 30°C were imaged for 10 h with pictures taken every 3 min, and 3-MB-PP1 treatment was added in the growth media used for imaging. Maximum-intensity projection of a 3 μm (0.75 μm step-size) is shown. Scale bar = 10 μm.
(AVI)

S11 Movie Growth of o/n treated FLCCR + DMSO cells in EMM. mCherry-Pcn1 (nuclear, magenta), mCherry-RitC (membrane, magenta), Sid2-GFP (cyan), and SynCut3-mTagBFP2 (green). Asynchronous growing cells at 30°C were imaged for 10 h with pictures taken every 3 min. Cells were pre-treated with DMSO o/n and DMSO treatment was added in the growth media used for imaging. Maximum-intensity projection of a 3 μm (0.75 μm step-size) is shown. Scale bar = 10 μm.
(AVI)

S12 Movie Growth of o/n treated FLCCR + 10 μm 3-MB-PP1 cells in EMM. mCherry-Pcn1 (nuclear, magenta), mCherry-RitC (membrane, magenta), Sid2-GFP (cyan), and SynCut3-mTagBFP2 (green). Asynchronous growing cells at 30°C were imaged for 10 h with pictures taken every 3 min. Cells were pre-treated with 10 μm 3-MB-PP1 o/n and 10 μm 3-MB-PP1 treatment was added in the growth media used for imaging. Maximum-intensity projection of a 3 μm (0.75 μm step-size) is shown. Scale bar = 10 μm.
(AVI)

S13 Movie Growth of o/n treated FLCCR *sty1-as* + DMSO cells in EMM. mCherry-Pcn1 (nuclear, magenta), mCherry-RitC (membrane, magenta), Sid2-GFP (cyan), and SynCut3-mTagBFP2 (green). Asynchronous growing cells at 30°C were imaged for 10 h with pictures taken every 3 min. Cells were pre-treated with DMSO o/n and DMSO treatment was added in the growth media used for imaging. Maximum-intensity projection of a 3 μm (0.75 μm step-size) is shown. Scale bar = 10 μm.
(AVI)

S14 Movie Growth of o/n treated FLCCR *sty1-as* + 10 μm 3-MB-PP1 cells in EMM. mCherry-Pcn1 (nuclear, magenta), mCherry-RitC (membrane, magenta), Sid2-GFP (cyan), and SynCut3-mTagBFP2 (green). Asynchronous growing cells at 30°C were imaged for 10 h with pictures taken every 3 min. Cells were pre-treated with 10 μm 3-MB-PP1 o/n and 10 μm 3-MB-PP1 treatment was added in the growth media used for imaging. Maximum-intensity projection of a 3 μm (0.75 μm step-size) is shown. Scale bar = 10 μm.
(AVI)

## Abbreviations

CDK: cyclin-dependent kinase
FLCCR: fluorescent-localized cell cycle reporter
FUCCI: fluorescence ubiquitin cell cycle indicator
PCR: polymerase chain reaction
TCA: trichloroacetic acid
