# Supplementary material for: FLCCR is a fluorescent reporter system that quantifies the duration of different cell cycle phases at the single-cell level in fission yeast
Source: PLoS Biol. 2025 Jan 7;23(1):e3002969. doi: 10.1371/journal.pbio.3002969 (PMC11706491; doi:10.1371/journal.pbio.3002969)
Supplement: S1 Table — (DOCX) [file pbio.3002969.s001.docx]

**S1 Table. List of yeast strains used in this work**

| **Strain** | **Genotype** | **Origen** |
| --- | --- | --- |
| WT | *972 h-* | [1] |
| AV18 | *h- sty1Δ::kanMX6* | Lab stock |
| AZ120 | *h- sty1-as (T97A)* | Lab stock |
| CS16 | *h- cdc25-22* | Lab stock |
| JA1512 | *h- rep2Δ::kanMX6* | Lab stock |
| JA3288 | *h+ sid2-GFP:ura4+ lys3::P.pcn1:mCherry-pcn1:lys3+ ade6::P.act1::mCherry-RitC::ade6+ leu1::P.eno101-SynCut3-mTagBFP2:leu1+ lys3-D20 ade6-D19 leu1-32 ura4-D18?* | This work |
| JA3319 | *h+ sty1Δ::kanMX6 sid2-GFP:ura4+ lys3::P.pcn1:mCherry-pcn1:lys3+ ade6::P.act1::mCherry-RitC::ade6+ leu1::P.eno101-SynCut3-mTagBFP2:leu1+ lys3-D20 ade6-D19 leu1-32 ura4-D18??* | This work |
| JA3417 | *h+ sty1-as (T97A) sid2-GFP:ura4+ lys3::P.pcn1:mCherry-pcn1:lys3+ ade6::P.act1::mCherry-RitC::ade6+ leu1::P.eno101-SynCut3-mTagBFP2:leu1+ lys3-D20 ade6-D19 leu1-32 ura4-D18?* | This work |
| JA3434 | *h+ cdr2Δ::kanMX6 sid2-GFP:ura4+ lys3::P.pcn1:mCherry-pcn1:lys3+ ade6::P.act1::mCherry-RitC::ade6+ leu1::P.eno101-SynCut3-mTagBFP2:leu1+ lys3-D20 ade6-D19 leu1-32 ura4-D18?* | This work |
| JA3446 | *h+ cdr1Δ::kanMX6 sid2-GFP:ura4+ lys3::P.pcn1:mCherry-pcn1:lys3+ ade6::P.act1::mCherry-RitC::ade6+ leu1::P.eno101-SynCut3-mTagBFP2:leu1+ lys3-D20 ade6-D19 leu1-32 ura4-D18?* | This work |
| JA3447 | *h+ rep2Δ::kanMX6 sid2-GFP:ura4+ lys3::P.pcn1:mCherry-pcn1:lys3+ ade6::P.act1::mCherry-RitC::ade6+ leu1::P.eno101-SynCut3-mTagBFP2:leu1+ lys3-D20 ade6-D19 leu1-32 ura4-D18?* | This work |
| JA3457 | *h? cdr1Δ::kanMX6* | This work |
| JA3458 | *h? cdr2Δ::kanMX6* | This work |
| JA3459 | *h? lys3::P.pcn1:mCherry-pcn1:lys3+ ade6::P.act1::mCherry-RitC::ade6+ leu1::P.eno101-SynCut3-mTagBFP2:leu1+is5:: P.pak1-CRIB(gic2aa2-181)-3GFP-NatMX:his5+ lys3-D20 ade6-D19 leu1-32 ura4-D18?* | This work |
| JA3499 | *h+ wee(5x) wee1::LEU2+ sid2-GFP:ura4+ lys3::P.pcn1:mCherry-pcn1:lys3+ ade6::P.act1::mCherry-RitC::ade6+ leu1::P.eno101-SynCut3-mTagBFP2:leu1+ lys3-D20 ade6-D19 leu1-32 ura4-D18?* | This work |
| JA3520 | *h+ sty1Δ::kanMX6 P.sty1:HA-atf1.10D::leu1 sid2-GFP:ura4+ lys3::P.pcn1:mCherry-pcn1:lys3+ ade6::P.act1::mCherry-RitC::ade6+ leu1::P.eno101-SynCut3-mTagBFP2:leu1+ lys3-D20 ade6-D19 leu1-32 ura4-D18? leu1::natMX6* | This work |
| JA3535 | *h+ cdc25-22 sid2-GFP:ura4+ lys3::P.pcn1:mCherry-pcn1:lys3+ ade6::P.act1::mCherry-RitC::ade6+ leu1::P.eno101-SynCut3-mTagBFP2:leu1+ lys3-D20 ade6-D19 leu1-32 ura4-D18?* | This work |
| JA3562 | *h- sty1Δ::kanMX6 sty1’::HA-atf1.10D::leu1 leu1-32* | This work |
| JA3651 | *h+ sty1-as (T97A) cdc25-22 sid2-GFP:ura4+ lys3::P.pcn1:mCherry-pcn1:lys3+ ade6::P.act1::mCherry-RitC::ade6+ leu1::P.eno101-SynCut3-mTagBFP2:leu1+ lys3-D20 ade6-D19 leu1-32 ura4-D18?* | This work |
| JA3654 | *h+ sty1-as (T97A) plo1-S402E sid2-GFP:ura4+ lys3::P.pcn1:mCherry-pcn1:lys3+ ade6::P.act1::mCherry-RitC::ade6+ leu1::P.eno101-SynCut3-mTagBFP2:leu1+ lys3-D20 ade6-D19 leu1-32 ura4-D18?* | This work |
| PN580 | *h- wee(5x) wee1::LEU2+ leu1-32 ade6-704* | [2] |

1. Leupold U. Genetical methods for *Schizosaccharomyces pombe*. Methods Cell Physiol. 4: D.M. Prescott; 1970. p. 169-77.

2. Russell P, Nurse P. The mitotic inducer nim1+ functions in a regulatory network of protein kinase homologs controlling the initiation of mitosis. Cell. 1987;49(4):569-76. Epub 1987/05/22. doi: 10.1016/0092-8674(87)90459-4. PubMed PMID: 3453113.
