# Supplementary material for: FLCCR is a fluorescent reporter system that quantifies the duration of different cell cycle phases at the single-cell level in fission yeast
Source: PLoS Biol. 2025 Jan 7;23(1):e3002969. doi: 10.1371/journal.pbio.3002969 (PMC11706491; doi:10.1371/journal.pbio.3002969)
Supplement: S2 Table — (DOCX) [file pbio.3002969.s002.docx]

**S2 Table. List of plasmids used in this work**

| **Plasmid** | **Description** | **Origin** |
| --- | --- | --- |
| JPp178 | pJK148-P.eno101:synCut3-mCherry:T.adh1 | [1] |
| p428.10D’ | pJK148-P.sty1:HA-Atf1.10D:T.nmt1 | [2] |
| pAV0357 | pLys3BstZ17I | [3] |
| pAV0757 | pHis5StuI-P.pak1:CRIB(gic2aa2-181)-3GFP:Scer\T.Adh1-kanMX | [3] |
| pAV0785 | pUra4AfeI-P.pcn1:mCherry-pcn1:T.nmt1-natMX | [3] |
| pAY1014 | pLSB-NatMX6 | [4] |
| pAY1035 | pLys3EcoNI-P.pcn1:mCherry-pcn1:T.nmt1-pTEV | This work |
| pAY1112 | pLSB-KanMX6 | [4] |
| pAY1193 | pJK148-P.eno101:synCut3-mTagBFP2:T.adh1 | This work |
| pAY1232 | pHis5StuI-P.pak1:CRIB(gic2aa2-181)-3GFP:Scer\Adh1-NatMX6 | This work |
| pAY1233 | pLSB-NatMX6 sty1-as sgRNA | This work |
| pAY1281 | pLSB-NatMX6 cdc25-22 sgRNA | This work |
| pAY1349 | pLSB-KanMX6 plo1-S402E sgRNA | This work |

1. Basu S, Greenwood J, Jones AW, Nurse P. Core control principles of the eukaryotic cell cycle. Nature. 2022;607(7918):381-6. Epub 2022/06/09. doi: 10.1038/s41586-022-04798-8. PubMed PMID: 35676478; PubMed Central PMCID: PMCPMC9279155.

2. Salat-Canela C, Paulo E, Sanchez-Mir L, Carmona M, Ayte J, Oliva B, et al. Deciphering the role of the signal- and Sty1 kinase-dependent phosphorylation of the stress-responsive transcription factor Atf1 on gene activation. J Biol Chem. 2017;292(33):13635-44. Epub 2017/06/28. doi: 10.1074/jbc.M117.794339. PubMed PMID: 28652406; PubMed Central PMCID: PMCPMC5566521.

3. Vjestica A, Marek M, Nkosi PJ, Merlini L, Liu G, Berard M, et al. A toolbox of stable integration vectors in the fission yeast Schizosaccharomyces pombe. J Cell Sci. 2020;133(1). Epub 2019/12/06. doi: 10.1242/jcs.240754. PubMed PMID: 31801797.

4. Torres-Garcia S, Di Pompeo L, Eivers L, Gaborieau B, White SA, Pidoux AL, et al. SpEDIT: A fast and efficient CRISPR/Cas9 method for fission yeast. Wellcome Open Res. 2020;5:274. Epub 2020/12/15. doi: 10.12688/wellcomeopenres.16405.1. PubMed PMID: 33313420; PubMed Central PMCID: PMCPMC7721064.
