# Supplementary material for: FLCCR is a fluorescent reporter system that quantifies the duration of different cell cycle phases at the single-cell level in fission yeast
Source: PLoS Biol. 2025 Jan 7;23(1):e3002969. doi: 10.1371/journal.pbio.3002969 (PMC11706491; doi:10.1371/journal.pbio.3002969)
Supplement: S3 Table — (DOCX) [file pbio.3002969.s003.docx]

**S3 Table. CRISPR oligonucleotides used in this work**

| **Oligo** | **Description** | **Sequence** |
| --- | --- | --- |
| J3049 | sty1-as sgRNA F | gactTGAAGATATTTATTTTGTCA |
| J3050 | sty1-as sgRNA R | aaacTGACAAAATAAATATCTTCA |
| J3051 | sty1 T97A HR forward T97A PAM | GGGAATTAAAACTATTAAAGCATTTAAGGCATGAGAATATTATTAGCTTGAGCGATATTTTTATTTCTCCCTTTGAAGATATTTATTTTGTCGCTGAGCT |
| J3052 | sty1 T97A HR forward T97A PAM | AGATTTGGTATAAGAAGTATTGTATAAATTGCGTCTCTAAAGGTCGCGATGTAAGTAGTCTATGAAGATCTGTTCCCAGAAGCTCAGCGACAAAATAAAT |
| J3177 | cdc25-22 sgRNA F | gactCGTAGTTAATTGGGTCACAA |
| J3178 | cdc25-22 sgRNA R | aaacTTGTGACCCAATTAACTACG |
| J3179 | cdc25-22 HR F PAM C532Y | TAGTCATCGTTATCCTTTCCTTTACTATCCCGAGGTTTATATACTTCATGGTGGTTACAAGTCGTTTTACGAAAACCACAAAAATAGATATGACCCAATT |
| J3180 | cdc25-22 HR R PAM C532Y | AAAGTAGCGTTTCGTTTGAAATTATTCATAGCCTTGGTGCAGGTCATAACATGCGAAGCATCGTTCATCGGAACGTAGTTAATTGGGTCATATCTATTTT |
| J3280 | plo1-S402E sgRNA F | gactTGAGCCTAAACTTCCATCAG |
| J3281 | plo1-S402E sgRNA R | aaacCTGATGGAAGTTTAGGCTCA |
| J3282 | plo1-S402E HR F S402E | GTGTCAACCCTGTTATGAAAATAGGACCGGAAACCAAACCTGTACCATCAAAGCTCTCTACAGCCCTCCATGCAGCTCGTAAAGAAACTGATGGAAGTTT |
| J3283 | plo1-S402E HR R S402E | GTTCAACTTGTTCAGTGACAGCGCTCTTAGTTGGAACAAATGATTGTGATTCTTCTCTCAAAACTTTAACTCTTGAGCCTAAACTTCCATCAGTTTCTTT |
