# Supplementary figures and images for: FLCCR is a fluorescent reporter system that quantifies the duration of different cell cycle phases at the single-cell level in fission yeast
Source: PLoS Biol. 2025 Jan 7;23(1):e3002969. doi: 10.1371/journal.pbio.3002969 (PMC11706491; doi:10.1371/journal.pbio.3002969)

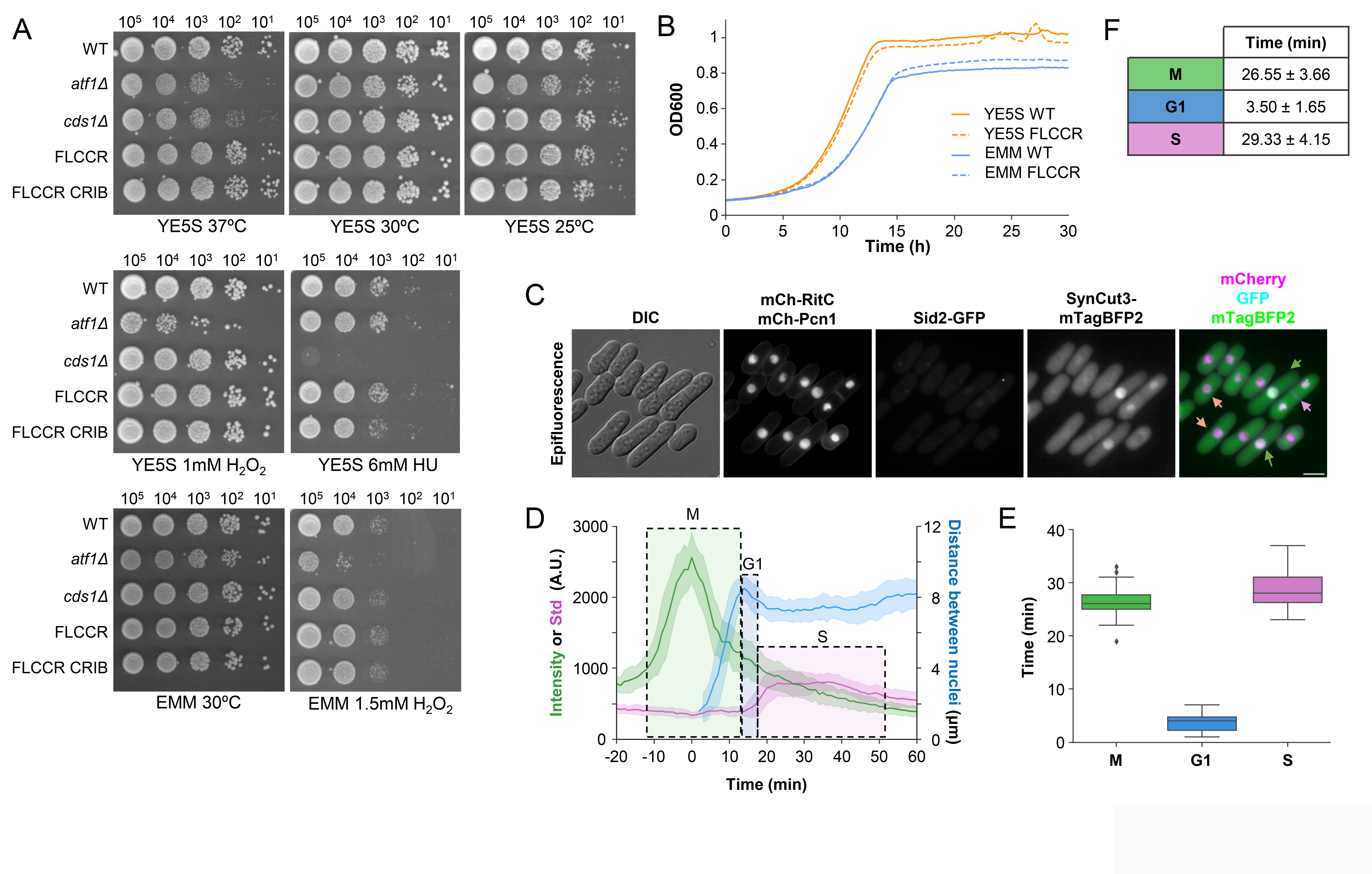

Supplement: S1 Fig — (A) Serial dilutions of the WT and FLCCR strains in YE5S or EMM plates. All plates were grown at 30°C, unless indicated. H2O2 or hydroxyurea (HU) were added at the indicated concentrations. (B) Growth curves comparing growth at 30°C of a WT and FLCCR strain in YE5S or EMM media. (C) Epifluorescence microscopy of the FLCCR strain. Arrows in merged image indicate a cell in S phase (magenta), G2 phase (salmon) or mitosis (green). Scale bar = 5 μm. (D) Nuclear SynCut3 mean fluorescence and Pcn1 Std fluorescence. Individual cycles were synchronized to peak SynCut3 nuclear fluorescence. To distinguish G1 from mitosis, we considered that mitosis was finished when the cell achieved the maximum distance between the 2 nuclei and G1 was defined as the time between the maximum separation of nuclei and entry into S phase. Green box marks mitosis, blue box marks G1 and magenta box marks DNA synthesis. n = 23 cells. (E, F) Quantification of the duration of the different cell cycle phases. Boxplot represents quartile distribution. Outliers are depicted as dots. Table shows mean ± standard deviation. n = 23 cells. (TIF) [file pbio.3002969.s004.tif]

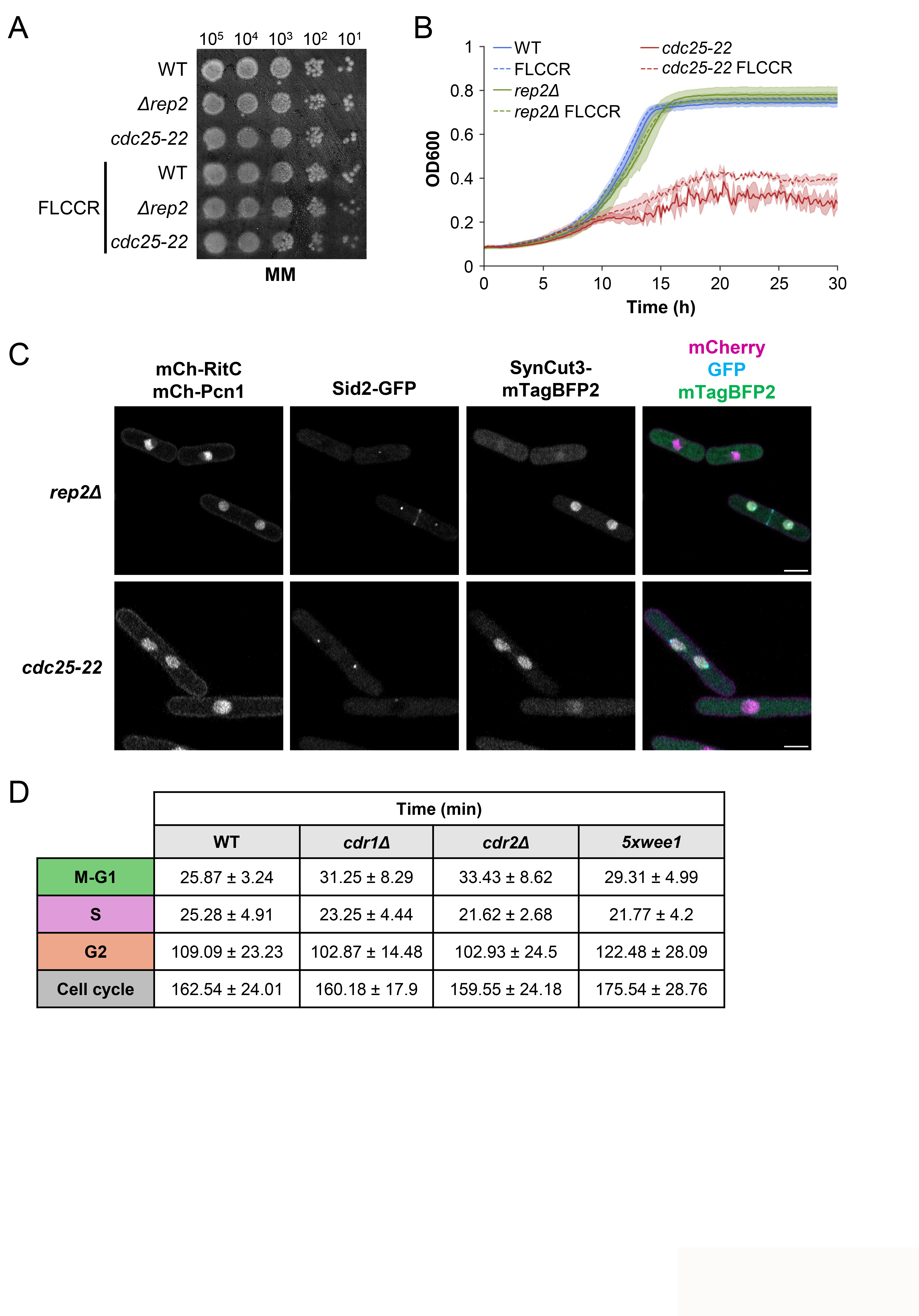

Supplement: S2 Fig — (A) Serial dilutions of the WT, rep2Δ and cdc25-22 strains in WT or FLCCR background. Plates were grown at 30°C for 3 days in minimal medium (MM). (B) Growth curves comparing growth at 30°C of a WT, rep2Δ and a cdc25-22 in WT or FLCCR backgrounds. n = 3 experiments. (C) Airyscan microscopy of a rep2Δ (top row) or a cdc25-22 (bottom row) in the cell cycle reporter background. Scale bar = 5 μm. (D) Quantification of the duration of the different cell cycle phases, shown as mean ± standard deviation. WT n = 224 cells; cdr1Δ n = 48 cells; cdr2Δ n = 58 cells; 5xwee1 n = 35 cells. (TIF) [file pbio.3002969.s005.tif]

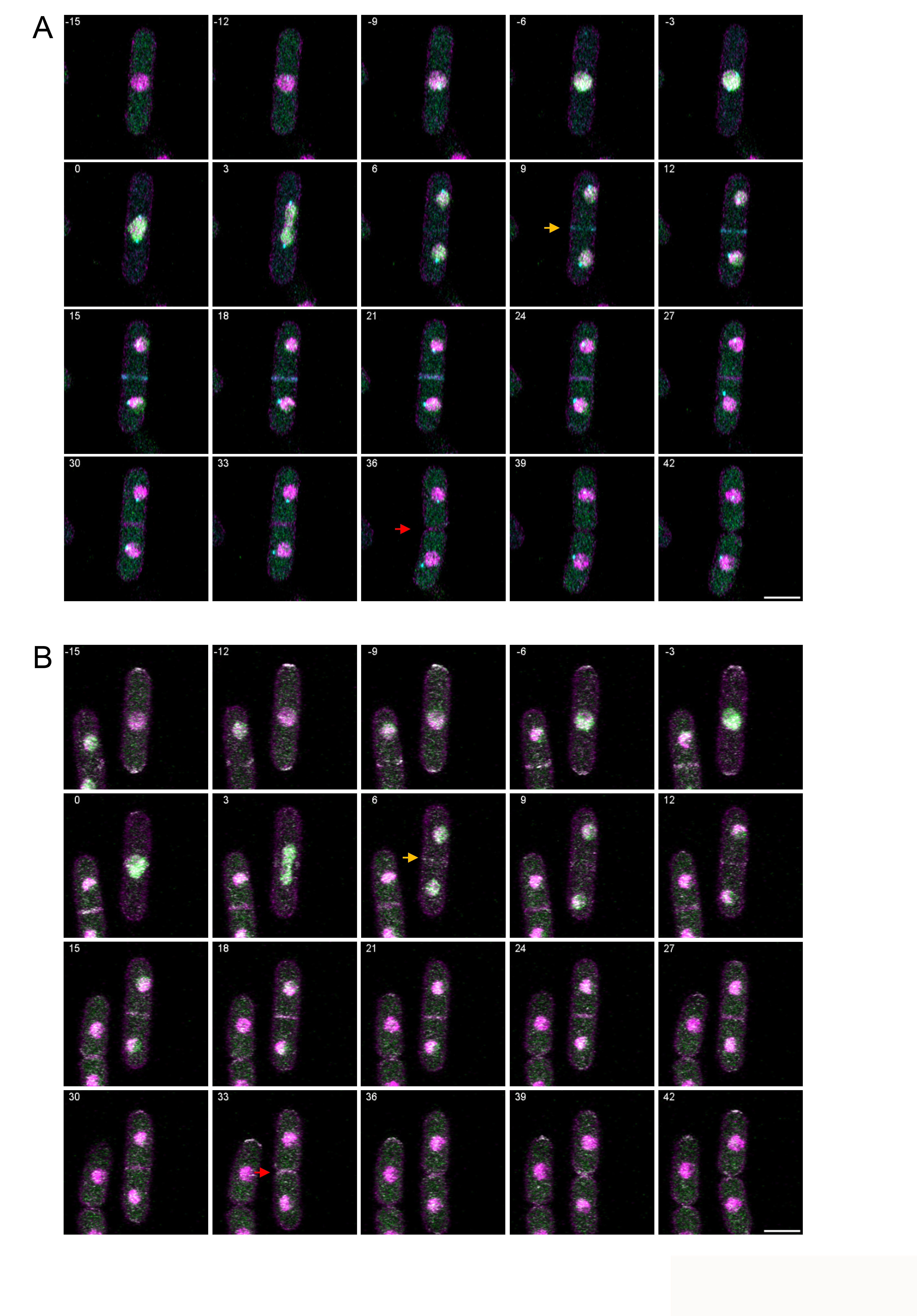

Supplement: S3 Fig — (A) Mitosis in a Sid2 FLCCR background. Time lapse between images is 3 min. Yellow arrow marks septation initiation and red arrow marks end of cytokinesis. Scale bar = 5 μm. (B) Mitosis in a CRIB combined with FLCCR. Time lapse between images is 3 min. Yellow arrow marks septation initiation and red arrow marks end of cytokinesis. Scale bar = 5 μm. (TIF) [file pbio.3002969.s006.tif]

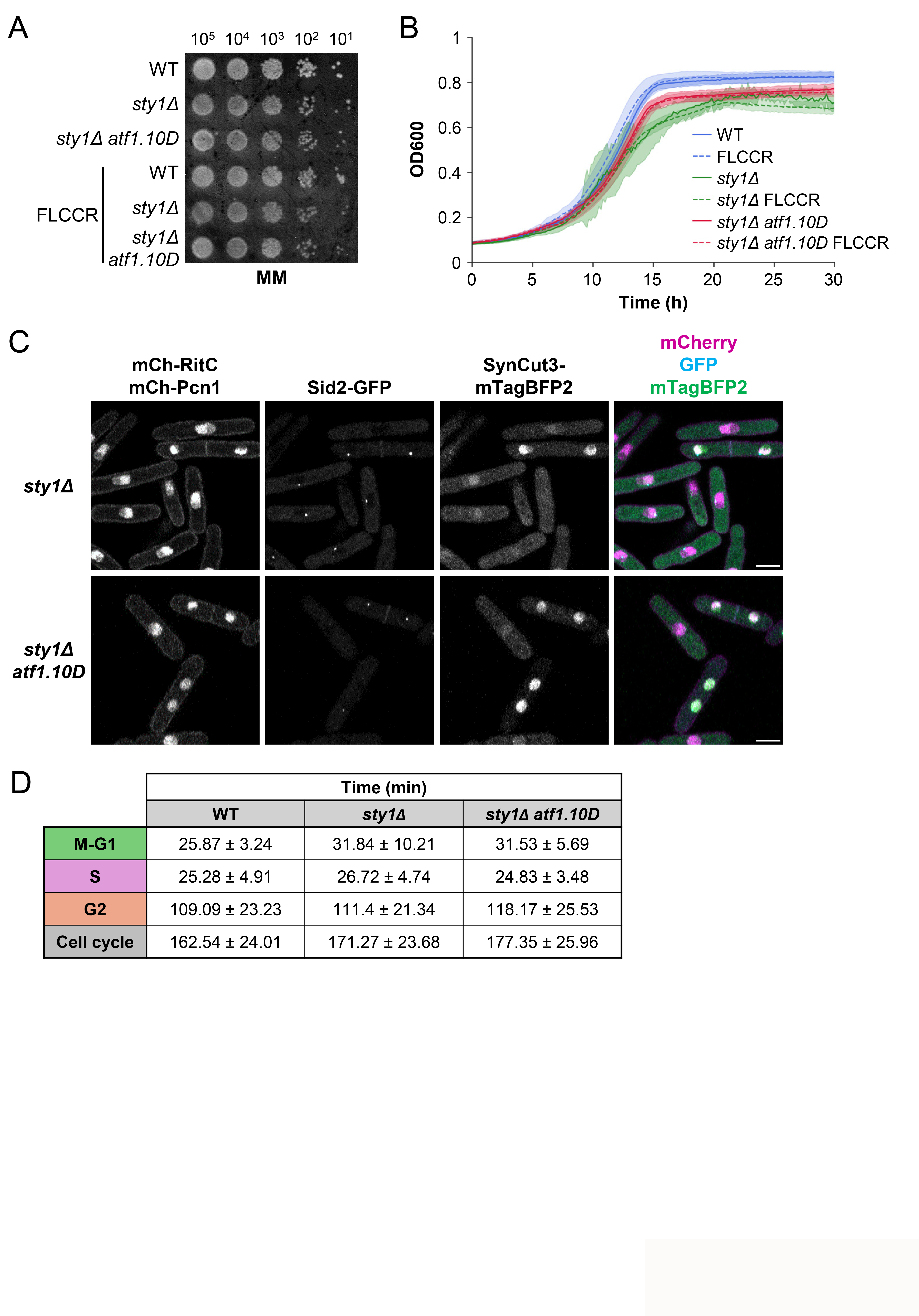

Supplement: S4 Fig — (A) Serial dilutions of the WT, sty1Δ and a sty1Δ atf1.10D strains in WT or FLCCR background. Plates were grown at 30°C for 3 days in minimal medium (MM). (B) Growth curves comparing growth at 30°C of a WT, sty1Δ and a sty1Δ atf1.10D in WT or FLCCR backgrounds. n = 4 experiments. (C) Airyscan microscopy of a sty1Δ and a sty1Δ atf1.10D in FLCCR background. Scale bar = 5 μm. (D) Quantification of the duration of the different cell cycle phases, shown as mean ± standard deviation. WT n = 224 cells; sty1Δ n = 43 cells; sty1Δ atf1.10D n = 65 cells. (TIF) [file pbio.3002969.s007.tif]

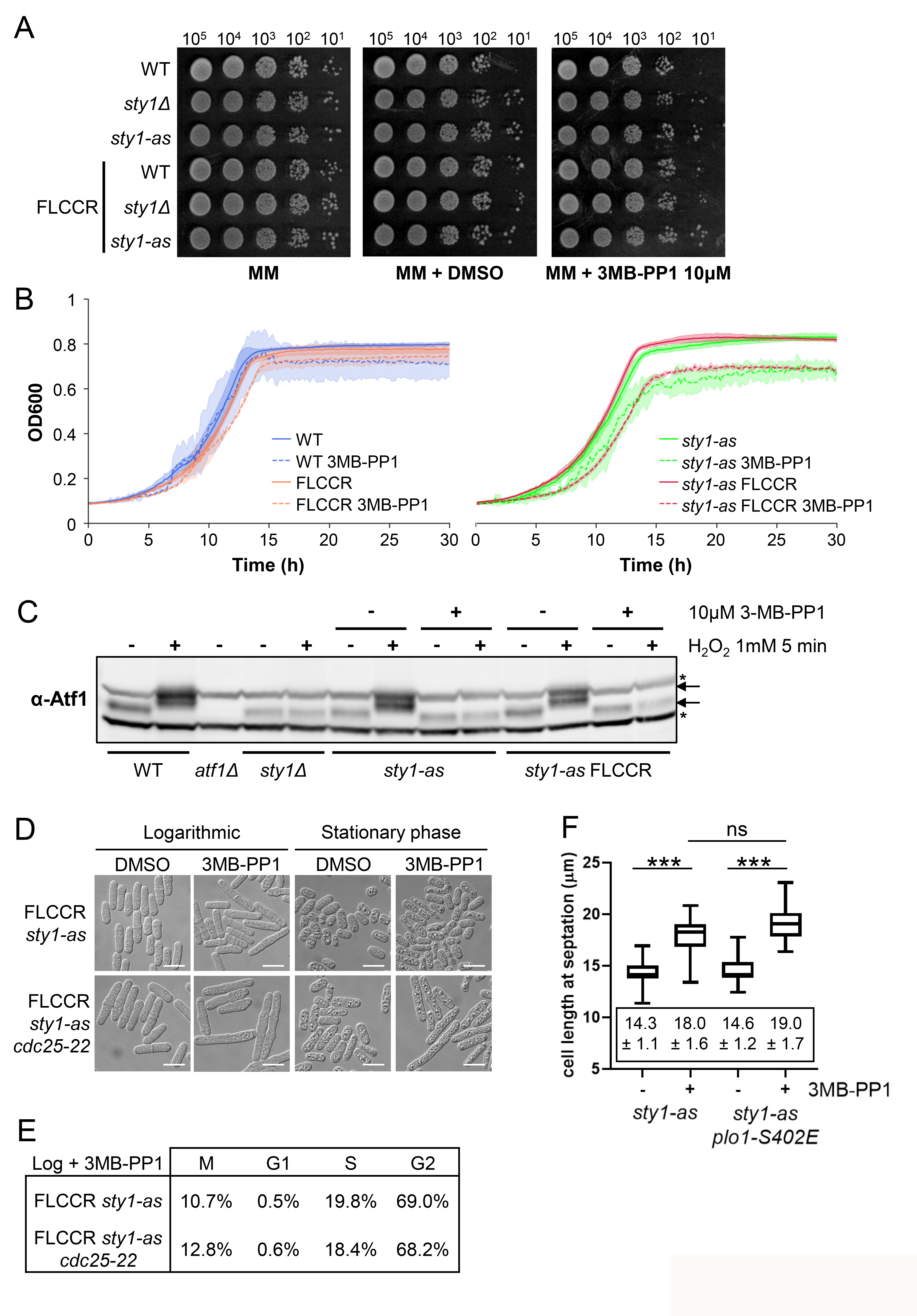

Supplement: S5 Fig — (A) Serial dilutions of the WT, sty1Δ and a sty1-as strains in WT or FLCCR background. Plates were grown at 30°C for 3 days in minimal medium (MM), with DMSO or with 10 μm 3MB-PP1. (B) Growth curves comparing growth at 30°C of a WT (left) and sty1-as (right) in WT or in the cell cycle reporter backgrounds in the presence of DMSO or 10 μm 3MB-PP1. n = 3. (C) Western blot α-Atf1 to detect Atf1 phosphorylation under oxidative stress conditions. sty1-as strain was pre-treated with 10 μm 3MB-PP1 for 15 min before addition of 1 mM H2O2. *, unspecific band; upper arrow points to the phosphorylated form of Atf1; lower arrow points to non-phosphorylated form of Atf1. (D) Representative DIC microscopy images of FLCCR sty1-as and FLCCR sty1-as cdc25-22 in logarithmic growth after 24 h of treatment with DMSO or 10 μm 3MB-PP1 or after 8 days in stationary phase with DMSO or 10 μm 3MB-PP1. Scale bar = 10 μm. (E) Table showing percentage (%) of cells in each cell cycle phase. FLCCR sty1-as (n = 187 cells) and FLCCR sty1-as cdc25-22 (n = 179 cells). (F) Boxplot showing cell length at septation of sty1-as and sty1-as plo1-S402E with DMSO or 3MB-PP1 10 μm treatment after 24 h. The inset indicates the mean ± SD. sty1-as DMSO n = 32 cells, sty1-as 3MB-PP1 n = 44 cells, sty1-as plo1-S402E DMSO n = 51 cells, sty1-as plo1-S402E 3MB-PP1 n = 50 cells. *** = p < 0.001. (TIF) [file pbio.3002969.s008.tif]

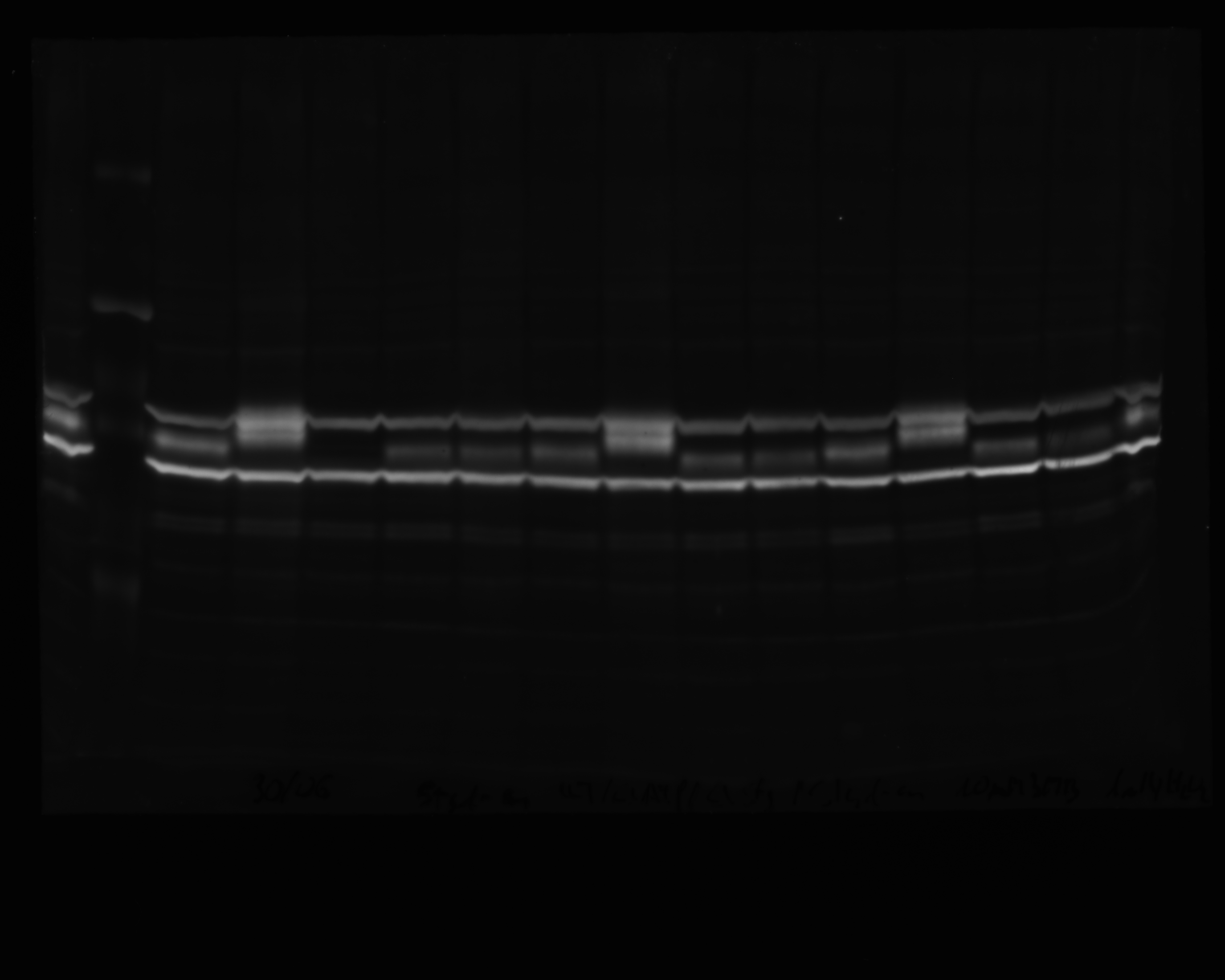

Supplement: S1 Raw Image — (TIF) [file pbio.3002969.s009.tif]
